# RNA-seq analysis of glycosylation related gene expression in STZ-induced diabetic rat kidney inner medulla

**DOI:** 10.3389/fphys.2015.00274

**Published:** 2015-10-01

**Authors:** Xiaoqian Qian, Xuechen Li, Titilayo O. Ilori, Janet D. Klein, Rebecca P. Hughey, Cong-jun Li, Abdel A. Alli, Zhengyu Guo, Peng Yu, Xiang Song, Guangping Chen

**Affiliations:** ^1^Department of Physiology, Emory University School of MedicineAtlanta, GA, USA; ^2^Department of Cardiology, The Fourth Affiliated Hospital of Harbin Medical UniversityHarbin, China; ^3^Renal Division, Department of Medicine, Emory University School of MedicineAtlanta, GA, USA; ^4^Renal-Electrolyte Division and Department of Cell Biology and Physiology, Department of Medicine, University of Pittsburgh School of MedicinePittsburgh, PA, USA; ^5^Bovine Functional Genomics Laboratory, United States Department of Agriculture - Agricultural Research ServiceBeltsville, MD, USA; ^6^Department of Electrical and Computer Engineering, TEES-AgriLife Center for Bioinformatics and Genomic Systems Engineering, Texas A&M UniversityCollege Station, TX, USA

**Keywords:** gene expression, Illumina, diabetes, urinary concentration, sialylation, fucosylation

## Abstract

The UT-A1 urea transporter is crucial to the kidney's ability to generate concentrated urine. Native UT-A1 from kidney inner medulla (IM) is a heavily glycosylated protein with two glycosylation forms of 97 and 117 kDa. In diabetes, UT-A1 protein abundance, particularly the 117 kD isoform, is significantly increased corresponding to an increased urea permeability in perfused IM collecting ducts, which plays an important role in preventing the osmotic diuresis caused by glucosuria. However, how the glycan carbohydrate structure change and the glycan related enzymes regulate kidney urea transport activity, particularly under diabetic condition, is largely unknown. In this study, using sugar-specific binding lectins, we found that the carbohydrate structure of UT-A1 is changed with increased amounts of sialic acid, fucose, and increased glycan branching under diabetic conditions. These changes were accompanied by altered UT-A1 association with the galectin proteins, β-galactoside glycan binding proteins. To explore the molecular basis of the alterations of glycan structures, the highly sensitive next generation sequencing (NGS) technology, Illumina RNA-seq, was employed to analyze genes involved in the process of UT-A1 glycosylation using streptozotocin (STZ)—induced diabetic rat kidney. Differential gene expression analysis combining with quantitative PCR revealed that expression of a number of important glycosylation related genes were changed under diabetic conditions. These genes include the glycosyltransferase genes Mgat4a, the sialylation enzymes St3gal1 and St3gal4 and glycan binding protein galectin-3, -5, -8, and -9. In contrast, although highly expressed in kidney IM, the glycosyltransferase genes Mgat1, Mgat2, and fucosyltransferase Fut8, did not show any changes. Conclusions: In diabetes, not only is UT-A1 protein abundance increased but the protein's glycan structure is also significantly changed. UT-A1 protein becomes highly sialylated, fucosylated and branched. Consistently, a number of crucial glycosylation related genes are changed under diabetic conditions. The alteration of these genes may contribute to changes in the UT-A1 glycan structure and therefore modulate kidney urea transport activity and alleviate osmotic diuresis caused by glucosuria in diabetes.

## Introduction

Urea is an important solute that contributes to the inner medullary osmolarity gradient in the kidney. The major mechanism for delivering urea to the inner medullary interstitium is urea reabsorption from the terminal inner medullary collecting duct (IMCD) mediated by the UT-A1 urea transporter. This process is mainly controlled by vasopressin *in vivo*. The importance of the urea transporter is evident in the UT-A1/UT-A3 knockout mouse, which demonstrates impaired urea clearance, reduced urinary concentration ability (Fenton et al., 2004), and hypertension (Jacob et al., 2008).

Native UT-A1 from kidney inner medulla (IM) is a heavily glycosylated protein with two glycosylated forms of 97 and 117 kDa; both are derived from a single 88-kDa core protein (Bradford et al., 2001; Chen et al., 2011). The 117-kDa form is fully glycosylated and contains N-glycans with poly-N-acetyllactosamine (poly-LacNAc) terminal processing, whereas the 97-kDa form is a hybrid form containing primarily the high mannose type of immature N-glycans (Chen et al., 2011). Interestingly, the 117 kDa form of UT-A1 increases dramatically in several states associated with decreased urea concentration, such as streptozotocin (STZ)-induced diabetes mellitus (Kim et al., 2003), a low-protein diet (Terris et al., 1998), hypercalcemia (Sands et al., 1998), water diuresis (Terris et al., 1998), and furosemide administration (Terris et al., 1998). A functional study using tubule perfusion showed that the increased 117 kDa glycoform in the IM is associated with increased urea transport activity (Pech et al., 2005). This suggests that changes in the relative abundance of the 97 and 117 kDa forms of UT-A1 may have important regulatory roles for UT-A1 function. Mutation of this protein revealed that N-linked glycosylation plays an important role in UT-A1 trafficking, protein stability and bioactivity (Chen et al., 2006). Loss of N-linked glycosylation significantly reduces urea transporter UT-A1 response to vasopressin (Chen et al., 2006). However, the underlying mechanism by which glycosylation affects these processes remains largely undetermined.

Asn (N)-linked glycosylation is generally a co-translational event that involves addition of a 14-sugar core oligosaccharide chain (Glc_3_Man_9_GlcNAc_2_) to consensus sites (Asn-X-Ser/Thr) within the nascent polypeptide in the endoplasmic reticulum (ER). Remodeling or maturation of the high mannose core oligosaccharide is a post-translational modification that occurs during transit of the glycoprotein from the ER through the Golgi complex and trans- Golgi network (TGN). Conversion of the high mannose core to a more complex type involves removal of glucose (Glc) and mannose (Man), and addition of sugars such as N-acetylglucosamine (GlcNAc), galactose (Gal), fucose (Fuc), and sialic acid. A specific enzyme carries out each step in the remodeling process, and the structure of a complex N-glycan on individual proteins can greatly vary among different tissues, organisms, and disease states due to the levels of enzyme expression. More than 200 enzymes participate in protein glycosylation and remodeling (Spiro, 2002; Nairn et al., 2008), thereby determining the abundance and diversity of individual N-glycan structures, making glycan maturation one of the most complex post translational modifications.

The recent development of next-generation RNA sequencing (RNA-Seq) technology provides a powerful method for profiling the entire transcriptome in small samples. Compared to hybridization-based methodologies of transcriptome analysis, RNA-Seq is not restricted to previously known transcripts and has low background, no hybridization bias, higher specificity, sensitivity, accuracy, and, importantly, provides quantitative information on mRNA transcript number (Wang et al., 2009; Hackett et al., 2012; Huber-Keener et al., 2012; Song et al., 2012). This new technology makes it suitable for us to analyze the molecular basis of UT-A1 glycan maturation both under physiological and pathophysiological situations.

In diabetes, kidney urea transport activity and urine concentration ability are markedly changed in response to increased glucose in urine and water loss (Kim et al., 2003; Pech et al., 2005). In the present study, using a differential sugar-specific binding protein—lectin, we discovered that the carbohydrate structure of UT-A1 is significantly changed under diabetic conditions. UT-A1 becomes highly sialylated, fucosylated, and branched. In addition, consistent with the alteration of glycan structure, UT-A1 binding to galectin proteins is also changed. To obtain transcript profiles of the glycan-modifying enzymes responsible for UT-A1 N-glycan structure changes in diabetes, we took advantage of Illumina mRNA sequencing (RNA-seq) technology and investigated gene expression in kidney IM from STZ-induced diabetic rats. Our goal of the RNA-seq analysis was to, at the transcript level, inspect whether the enzymes involved in the glycosylation processes of sialylation, fucosylation, glycan chain branching and glycan binding to galectin proteins, were altered in normal and diabetic kidney.

## Materials and methods

### STZ rat models

Male Sprague-Dawley rats, 3 months (~150 g), were purchased from Charles River Laboratories. We used the well-established streptozotocin (STZ)-induced diabetic rat model (Kim et al., 2003; Chen et al., 2011). Rats were injected with STZ (62.5 mg/kg body weight prepared fresh in 0.1 M citrate buffer, pH 4.0) or vehicle into the tail vein. Diabetes was confirmed by measuring blood glucose (One Touch Profile Diabetes Tracking Kit) 24 h after STZ injection. At 15 d after injection, rats were sacrificed by decapitation. The terminal half of the IM (IM tip) was dissected from kidney and used for protein and RNA preparation. All animal protocols were approved by the Emory University Institutional Animal Care and Use Committee (IACUC).

### Lipid raft preparation and lectin pulldown

Cell membrane lipid raft fractions were used for lectin pulldown experiments. Lipid rafts from freshly isolated IM tip were prepared with a 5–40% sucrose discontinuous gradient ultracentrifugation protocol as described (Huang et al., 2010; Chen et al., 2011). Equal amounts of pooled lipid raft fractions (fractions 2–4) were incubated with 30 μl of an agarose-conjugated lectin suspension at 4°C overnight. After washing, the precipitated proteins were subjected to Western blotting with UT-A1 antibody. All the agarose-conjugated lectins were purchased from Vector Laboratories. The lectins used in this study are *concanavalin* A (Con A), *galanthus nivalis lectin* (GNL), wheat germ agglutinin (WGA), tomato lectin, *datura stramonium lectin* (DSL), *phaseolus vulgaris leukoagglutinin* (PHA-L), *sambucus nigra lectin* (SNA), *maackia amurensis lectin II* (MAL II), and *aleuria aurantia lectin* (AAL).

### GST-galectin fusion protein preparation and pulldown

The cDNAs for Gal-1, -3, -4, -7, -8, -9N, and -9C were subcloned into the bacterial vector pGEX-6P-1 and the GST fusion galectin proteins were prepared from bacteria as described (Poland et al., 2011). The fusion proteins were first affinity purified on glutathione-conjugated Sepharose and then lactose-conjugated Sepharose to insure that galectins were active. Equal amount of cell membrane fractions from rat IM were incubated with freshly prepared GST-galectin proteins bound to glutathione-conjugated beads for 1 h at 4°C. The beads were washed with buffer A (0.05 M Tris-HCl, pH 8, 150 mM NaCl) containing 14 mM β-mercaptoethanol, and then sequentially eluted with buffer containing 0.1 M sucrose (negative control) and 0.1 M lactose (specific-binding). The eluted proteins were analyzed by Western blot with anti-UT-A1 antibody. NIH ImageJ software was used to quantify the band density from three independent experiments. Data were expressed as mean ± SD. Statistical analysis of the data was performed by One-Way ANOVA. Differences were considered as significant at ^*^P < 0.05 or ^**^P < 0.01.

### IM tip RNA isolation and cDNA synthesis

For the RNA-seq study, the total RNA from rat IM tip was extracted using Trizol Reagent (Invitrogen). The RNA samples were purified using an RNeasy Mini Kit (Qiagen). Quantification and purity assessment of the RNA samples were determined on a NanoDrop Spectrophotometer (Nano-Drop Technologies). RNA quality was assessed with an Agilent Bioanalyzer 2100. Equal amounts of purified mRNA was transcribed to cDNA using a SMARTer PCR cDNA Synthesis Kit (Clontech Cat#634925).

### Library preparation and illumina HiSeq2000 sequencing

The cDNAs (Ctrl *n* = 3, STZ *n* = 3) for high-throughput sequencing were fragmented by DNase I and ligated to Illumina adapters. These adapter-ligated cDNA fragments were amplified and sequenced on the Illumina HiSeq2000 sequencer.

### RNA-seq data processing

Raw sequence reads from the FASTQ files from six samples were mapped against rat reference genome rn4 with STAR2.3.1t (Dobin et al., 2013). Only the uniquely mapped reads were used to calculate the numbers of reads per gene. The counts of the control group and the STZ group were tabulated in a table. This table was then fed to DESeq (Anders and Huber, 2010) for normalization and identification of differentially expressed genes between these two groups using the standard workflow. To correct for multiple hypothesis testing, the Benjamini–Hochberg procedure was used with an FDR cutoff of 0.05. Functional category and pathway analysis of diabetes-dependent changed genes were performed using IPA (Ingenuity Pathways Analysis, www.ingenuity.com). Unfortunately, the IPA analysis did not pick up the glycosylation-related genes; either the data set does not have such genes or the IPA may not have glycosylation pathways. We therefore manually searched and summarized those genes involving glycosylation process of sialylation, fucosylation, glycan chain branching, and glycan binding protein galectins from the RNA-seq data.

### Quantitative real time PCR (qRT-PCR)

Quantitative real-time PCR were performed as we described before (Chen et al., 2010). The complementary DNAs from total RNA samples were synthesized by reverse transcription (RT) with SuperScript reverse transcriptase (BD Bioscience). Gene-specific primers were designed to generate amplicons of length 100–250 nucleotides by using the Invitrogen Primer program. Prior to real-time PCR, a single amplified product of the expected size was confirmed by regular PCR and gel electrophoresis. All amplified products were subcloned into TA vector and further verified by DNA sequencing. Real-time PCR were carried out using the Bio-Rad iCycler Real-Time Detection System with a three-step protocol. Cycling conditions were set as 95°C for 3 min, followed by 40 cycles of 30 s at 95°C, 30 s at 55°C, and 30 s at 72°C. Fluorescence of the amplificates was detected with the iQTM SYBR Green Supermix (Bio-Rad). Data were normalized using the ratio of GAPDH and analyzed by iCycler software3.0 (Bio-Rad). Primers specific for each of the genes are shown in Supplemental Table S4. Significance was determined using a Student's *t*-test for each targeted gene.

## Results

### UT-A1 is highly sialylated, fucosylated, and branched in diabetes

**Figure 1:**
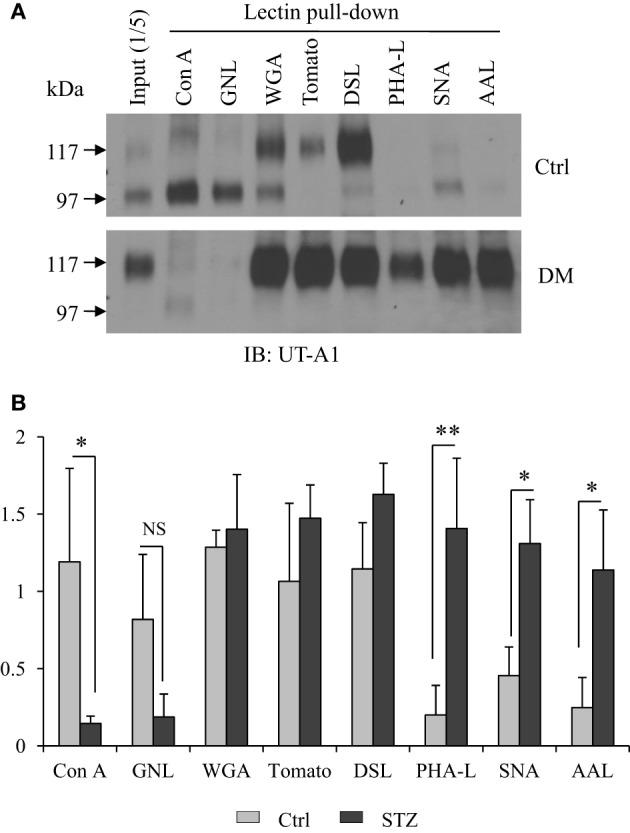
**Lectin pulldown assays. (A)** Cell membrane lipid raft fractions isolated from control or STZ-diabetic rat IM tip were pooled and incubated with 30 μl of indicated agarose-bound lectins at 4°C overnight. After washing, the lectin precipitated samples were analyzed by immunoblotting with anti-UT-A1 antibody. **(B)** Densitometry analysis of UT-A1 protein bands from three separate experiments (*n* = 3). Each lectin precipitated UT-A1 was normalized with UT-A1 from input proteins (means ± SD, ^*^P < 0.05, ^**^P < 0.01, NS, no significance).

Cell membrane UT-A1 is primarily localized in lipid raft microdomains (Chen et al., 2011). To assess differences in the N-glycan structure of UT-A1 in control and STZ rat IM, we isolated lipid raft membrane fractions as previously described and used this material for lectin binding assays as described in Materials and Methods. As shown in Figure 1A, the 97 kDa form of UT-A1 from control rat IM rafts bound primarily to Con A and GNL (mannose-specific) and WGA (GlcNAc specific), but was not detected in the GNL or Con A bound fractions of the STZ rats. The 117 kDa form of UT-A1 bound primarily to WGA (GlcNAc specific), tomato lectin (poly-N-acetyllactosamine), and DSL (repeating N-acetyllactosamine). Interestingly, the proportion of UT-A1 bound to SNA, AAL, and PHA-L form in the STZ rat IM is notably increased when compared to the proportion in the control rat IM. The increased UT-A1 binding to SNA, AAL, and PHA-L is not due to the protein overloading or extended exposure time since the UT-A1 bound to Con A and GNL is not increased. On the contrary, there is a decrease of the lower glycosylated 97 kDa UT-A1 pulled down by Con A and GNL under diabetic situations. Binding of 117 kDa UT-A1 to SNA, AAL, and PHA-L is consistent with the presence of sialic acid, fucose, and tetra-antennary branched glycans, respectively. Figure 1B is the densitometry analysis of lectin bound UT-A1 of control and STZ rat IM samples from three independent experiments.

### Increased association of galectin-3, 7, 8, 9 with 117 kDa UT-A1 in diabetes

**Figure 2:**
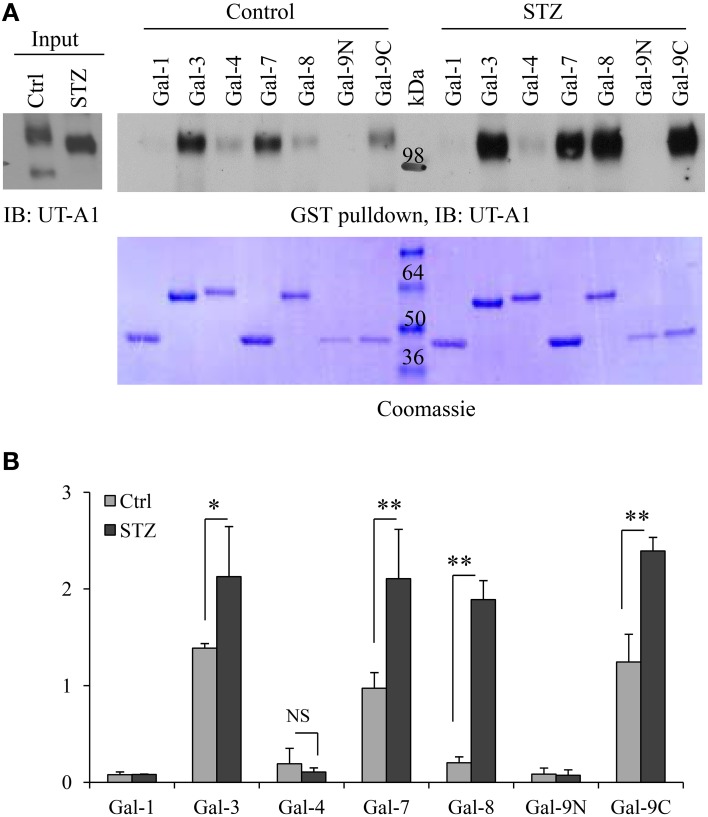
**GST-galectin pulldown assays. (A)** Equal amount of cell membrane fractions from rat IM tip were incubated with freshly prepared GST-galectin proteins for 1 h at 4°C. After washing with buffer A containing 14 mM β-mercaptoethanol and buffer containing 0.1 M sucrose, the galectin specific binding proteins were eluted by 0.1 M lactose (specific-binding) and subsequently analyzed by Western blot with UT-A1 antibody (Top). The same membrane was stained by Coomassie brilliant blue to verify GST fusion galectin proteins (Bottom). **(B)** Densitometry analysis of UT-A1 protein bands from three separate experiments (*n* = 3). Each GST-galectin precipitated UT-A1 was normalized with UT-A1 from input (means ± SD, ^*^P < 0.05, ^**^P < 0.01, NS, no significance).

Galectins are a group of small lectin-like proteins (14–30 kDa) that bind β-galactose-containing glycoconjugates. Each galectin has unique binding specificities (Poland et al., 2011). To investigate whether the change of the UT-A1 glycan structure under diabetic conditions would result in alteration of UT-A1 binding to galectin proteins, we performed the GST-galectin pulldown assay with control and STZ rat IM samples as described in Materials and Methods. GST-galectin proteins pre-bound to glutathione beads were incubated with equal amounts of lipid raft membrane fractions from kidney IM and eluted with lactose. The binding of UT-A1 was examined by Western blot analysis of the eluted material. Galectin proteins are predicted to bind only the high glycosylation form of 117 kDa, as the 97 kDa form exhibits only immature high mannose Gal-deficient N-glycans (Chen et al., 2011 and Figure 1). As shown in Figure 2A, we found that the 117 kDa form of UT-A1 from control rat IM binds to primarily Gal-3 and Gal-7 with a small amount binding to Gal-8 and Gal-9C. However, we observed increased binding of the 117 kDa UT-A1 to Gal-3, -7, -8, and -9, particularly the enhanced binding to Gal-8 and -9 indicating that the N-glycans are changed on UT-A1 in diabetic rat kidney. The increased UT-A1 bound to Gal-3, -7, -8, and -9 is not because of protein overloading since UT-A1 bound to Gal-1 and -4 is not increased. Figure 2B shows the signal quantification and statistical analysis from three independent studies. Galectin-9 has two carbohydrate recognition domains (CRD). Since GST-Gal-9 is aggregated in the bacteria, we prepared N-terminal and C-terminal CRDs separately as GST-Gal-9N (residues 1–148) and GST-Gal-9C (residues 225–355) (Poland et al., 2011). Only C-terminal, but not N-terminal, CRD in galectin-9 interacted with UT-A1. Additionally, we observed that the 117 kDa form of UT-A1 from diabetic tissue migrates further upon electrophoresis, reflecting different glycosylation modifications occurred in the diabetic animal.

### General RNA-seq results

In order to reveal potential genes and mechanisms that are involved in UT-A1 glycosylation modification, we performed the RNA-Seq by using RNA samples from control (*n* = 3) and STZ rat (*n* = 3) IM tip. RNA-Seq generated an average of 14.2 ± 1.44 (mean ± SD) million reads per sample (Supplemental Table S1). About 50 ~ 70% of the reads were mapped to the rat reference genome rn4 by STAR. Among a total 25,809 genes annotated in Ensembl RGSC3.4, 8197 genes had enough reads and therefore were selected for DESeq analysis, which determined 552 genes as significantly different (FDR < 0.05) between the control and STZ rats. Supplemental Figure S1 shows the volcano plot analysis of differentially expressed genes in normal and STZ rat IM. Table 1 lists the top 15 most significantly up- or down-regulated genes by log_2_ fold change in kidney IM tip under diabetic condition.

**Table 1:** **The top 15 up- and down-regulated genes in STZ rat IM by RNA-seq analysis**.

**Gene**	**Description**	**Fold change (log_2_)**	***p*-value**	**FDR**
**UP-REGULATED GENES**				
Ctse	Cathepsin E	4.9	4.01E-55	3.29E-51
Wfdc6b	WAP four-disulfide core domain 6B	4.8	8.37E-25	1.37E-21
Lypd2	Ly6/Plaur domain containing 2	4.4	6.92E-22	7.09E19
Pon3	Paraoxonase 3	4.1	1.98E-23	2.32E-20
RGD1563047	BPI fold containing family B, member 1	3.4	9.05E-06	5.31E-04
Fam111a	Family with sequence similarity 111	3.4	5.77E-19	3.15E-16
St3gal4	ST3 beta-galactoside alpha-2,3-sialyltransferase 1	3.4	4.17E-30	1.14E-26
Upk3a	Uroplakin 3A	3.3	9.11E-17	3.93E-14
Wfdc10	WAP four-disulfide core domain 10	3.3	3.87E-10	7.56E-08
Slc13a2	Solute carrier family 13	2.8	2.88E-21	2.62E-18
Oas1f	2′–5′ oligoadenylate synthetase 1F	2.8	2.59E-05	1.27E-03
RGD1564463	Similar to Mdes protein	2.8	3.82E-06	2.70E-04
Rarres2	Retinoic acid receptor responder 2	2.7	1.49E-23	2.03E-20
Acer2	Alkaline ceramidase 2	2.7	3.14E-03	4.74E-02
Alox15b	Arachidonate 15-lipoxygenase, type B	2.5	1.10E-06	8.93E-05
**DOWN-REGULATED GENES**				
Ypel4	Yippee-like 4 (Drosophila)	−5.1	2.68E-06	1.96E-04
Umod	Uromodulin	−3.1	4.92E-27	1.01E-23
Ccl7	Chemokine (C-C motif) ligand 7	−3.1	1.61E-36	6.60E-33
Mucdhl	Mucin and cadherin like	−2.6	2.24E-08	3.01E-06
Ifit1	IFN-induced protein with tetratricopeptide repeats 1	−2.5	2.77E-14	9.47E-12
Ifit3	IFN-induced protein with tetratricopeptide repeats 3	−2.5	5.67E-06	3.66E-04
Loxl4	Lysyl oxidase-like 4	−2.5	4.26E-21	3.18E-18
Tff3	Trefoil factor 3, intestinal	−2.4	9.13E-07	7.87E-05
Kap	Kidney androgen regulated protein	−2.2	4.71E-05	2.04E-03
Ifit2	IFN-induced protein with tetratricopeptide repeats 2	−2.2	1.27E-06	1.02E-04
S100a5	S100 calcium binding protein A5	−2.1	2.10E-16	8.60E-14
Egf	Epidermal growth factor	−2.1	1.32E-13	4.01E-11
Mgp	Matrix Gla protein	−2.1	8.08E-11	1.84E-08
Zcchc12	Zinc finger, CCHC domain containing 12	−2.1	2.30E-09	3.70E-07
Gpnmb	Glycoprotein (transmembrane)	−2.0	6.26E-11	1.46E-08

Functional category and pathway analysis of diabetes-dependent changed genes were further evaluated by using IPA (Ingenuity Pathways Analysis, www.ingenuity.com). The upregulated and downregulated networks under diabetes are presented in Supplemental Tables S2, S3. Unfortunately, the IPA analysis did not pick up the glycosylation-related genes, either the data set does not have such genes or the IPA may not have glycosylation pathways. We therefore manually searched and summarized those genes from RNA-seq data involved in sialylation, fucosylation, glycan chain branching, and glycan binding protein galectins.

### The Mgat family of acetylglucosaminyltransferases

GlcNActransferase (Mgat) enzymes belong to the family of glycosyltransferases. By the addition of N-acetylglucosamine to the N-linked sugar chains, Mgat enzymes catalyze the formation of tri- and multi-antennary branching structures in the Golgi apparatus (Rini et al., 2009; Stanley et al., 2009). As shown in Figure 1, UT-A1 from diabetic rats undergoes increased glycan branching, indicated by its affinity to lectin PHA-L. We examined N-glycan branching enzyme Mgat expression in the kidney. Table 2 lists the Mgat genes expressed in kidney IM according to the RNA-seq analysis. Three Mgat enzymes (Mgat1 > Mgat2 > Mgat4a) were highly expressed in kidney IM. However, the transcripts of Mgat gene in the kidney did not change, indicating that changes in UT-A1 glycan branching may not be due to changes in Mgat transcription levels. Mgat3, Mgat4b, Mgat4c, Mgat5, and Mgat5b were not expressed (or are expressed at very low levels) in kidney IM.

**Table 2:** **Summary of acetylglucosaminyltransferase genes from kidney IM by RNA-seq analysis**.

**Gene ID**	**Gene name**	**Average counts**		***p*-value**	**FRD**
		**Ctrl**	**STZ**		
Mgat1	Mannoside acetylglucosaminyltransferase 1	118.0	109.5	0.777	0.945
Mgat2	Mannoside acetylglucosaminyltransferase 2	76.5	103.3	0.127	0.454
Mgat3	Mannoside acetyl glucosaminyltransferase 3	NA^*^	NA		
Mgat4a	Mannoside acetylglucosaminyltransferase 4, isoenzyme A	48.1	35.8	0.258	0.629
Mgat4b	Mannoside acetylglucosaminyltransferase 4, isoenzyme B	NA	NA		
Mgat4c	Mannosyl (α-1,3-)-glycoprotein β-1,4-N- acetylglucosaminyltransferase, isozyme C	NA	NA		
Mgat5	Mannoside acetylglucosaminyltransferase 5	NA	NA		
Mgat5b	Mannoside acetylglucosaminyltransferase 5, isoenzyme B	NA	NA		

* *Indicate no or very low non-reliable calculation*.

### Sialyltransferases and neuraminidases

As reported before (Chen et al., 2011) and shown in the current study (Figure 1), UT-A1 contains a high amount of SNA-bound sialic acid in its glycan structure under diabetic situations (Figure 1). We recently reported that modification with sialic acid increases UT-A1 membrane stability and urea transport activity (Li et al., 2014). Here, we explored sialylation related enzymes in diabetes. There are three categories of sialyltransferases. In the type I enzyme family of α-2, 3 sialyltransferases, St3gal (ST3 β-galactoside α-2, 3-sialyltransferase) variants 2, 4, and 6 are found in kidney IM (Table 3). The most prominently expressed α-2, 3 sialyltransferase in the kidney IM region is St3gal6, however it did not change under the diabetic condition. The most significant increased sialyltransferase is St3gal4 (*q* = 1.14E-26) in diabetes. There is no change for St3gal2 and ST3gal6. In the type II enzyme family of α-2, 6 sialyltransferases, ST6gal1, St6galnac2, and 3 are highly expressed in kidney IM but they did not change in diabetes. Clearly, RNA-seq analysis showed that kidney IM does not express (or expresses at very low levels) the members of the type III enzyme family of α-2,8 sialyltransferases.

**Table 3:** **Summary of sialyltransferase and neuraminidase genes from kidney IM**.

**Category**	**Gene name**	**Average counts**		***p*-value**	**FRD**
		**Ctrl**	**STZ**		
α2,3 sialyltransferase	St3gal1	NA	NA		
	St3gal2	33.1	38.2	0.623	0.886
	St3gal3	NA	NA		
	St3gal4	52.8	542.5	4.17E-30	1.14E-26
	St3gal5	NA	NA		
	St3gal6	289.4	289.2	0.982	1.000
α2,6 sialyltransferase	St6gal1	166.5	125.8	0.108	0.416
	St6galnac1	NA	NA		
	St6galnac2	34.4	45.3	0.317	0.685
	St6galnac3	58.6	82.8	0.088	0.374
α2,8-sialyltransferase	St8sia1	NA	NA		
	St8sia2	NA	NA		
	St8sia3	NA	NA		
	St8sia4	NA	NA		
	St8sia5	NA	NA		
	St8sia6	NA	NA		
Neuraminidase (Sialidase)	Neu1	6.4	13.6	0.146	0.999
	Neu2	NA	NA		
	Neu3	NA	NA		
	Neu4	NA	NA		

*St3gal: ST3 β-galactoside α-2,3-sialyltransferase*.

*St6galnac: ST6 (α-N-acetyl-neuraminyl-2,3-β-galactosyl-1,3)-N-acetylgalactosaminide α-2,6-sialyltransferase*.

*St8sia: ST8 α-N-acetyl-neuraminide α-2,8-sialyltransferase*.

Glycoprotein sialylation is reversely regulated by neuraminidase (sialidase). Sialidases hydrolyze terminal sialic residues in glycoproteins and reduce protein sialylation. There are four neuraminidases (Neu). RNA-Seq analysis revealed that only Neu1 is expressed in kidney IM. The Neu1 enzyme transcript shows an increasing tendency but no significant increases under diabetic conditions (Table 3).

### Glycoprotein fucosylation and fucosyltransferases

Lectin pulldown assay in Figure 1 showed an increase of UT-A1 fucosylation pulled down by AAL in diabetes. Glycoprotein fucosylation is mediated by fucosyltransferases. There are more than eight fucosyltransferases in mammals. Surprisingly, RNA-Seq analysis (Table 4) shows that the kidney IM only expresses the α-1,6 fucosyltransferase 8 (Fut 8) mRNA isoform. Fut 8 catalyzes fucose residue transfer within the Golgi apparatus from GDP-fucose to the innermost GlcNAc residue of hybrid and complex N-glycan via α 1,6-linkage (termed core fucosylation) (Nishikawa et al., 1992). The Fut 8 transcript is decreased but does not reach statistical significance in diabetes. Kidney IM does not express the peripheral α-1,3-fucose enzymes of Fut1, 2, 4, 7, 9, 10, and 11.

**Table 4:** **Summary of fucosyltransferase genes from kidney IM by RNA-seq analysis**.

**Gene ID**	**Gene name**	**Average counts**		***p*-value**	**FRD**
		**Ctrl**	**STZ**		
**ALPHA (1,3) FUCOSYLTRANSFERASE**					
Fut1	Fucosyltransferase 1	NA	NA		
Fut2	Fucosyltransferase 2	NA	NA		
Fut4	Fucosyltransferase 4	NA	NA		
Fut7	Fucosyltransferase 7	NA	NA		
Fut9	Fucosyltransferase 9	NA	NA		
Fut10	Fucosyltransferase 10	NA	NA		
Fut11	Fucosyltransferase 11	NA	NA		
**ALPHA (1,6) FUCOSYLTRANSFERASE**					
Fut8	Fucosyltransferase 8	76.0	51.8	0.061	0.313

### Glycan binding protein galectin

The galectin family is defined by having at least one characteristic CRD with an affinity for beta-galactosides (Rabinovich et al., 2007). Carbohydrate structure changes often affect a glycoprotein's binding affinity for the galectin proteins. By their association with glycans, galectin proteins play many important roles in regulating glycoprotein function. There have been 15 galectins discovered in mammals, encoded by the LGALS genes (Cummings and Liu, 2009). Galectins are divided in three categories based on their distinct structures: the prototypical, chimera, and tandem galectins. RNA-seq analysis showed four types of galectin genes are expressed in a high abundance in kidney IM, Gal-9 > Gal-1 > Gal-3 >> Gal-5 (Table 5). Galectin-1 is highly expressed galectin in kidney IM, however GST pull-down assay did not show its association with UT-A1 (Figure 1). Moreover, galectin-1 expression did not change in diabetes. Therefore, galectin-1 is unlikely to regulate UT-A1. Galectin-9 is abundantly expressed in kidney but its expression is significantly decreased (*p* = 0.001, *q* = 0.019) under diabetic conditions. Galectin-3 is unique and is the only member of the chimera galectin group, designated as such because it has an extended N-terminus. Figure 2 shows galectin-3 is the major galectin that binds to UT-A1 under non-stimulated conditions; this binding is increased in diabetes. In agreement with the increased galectin-3 and UT-A1 binding activity, galectin-3 gene expression is increased about ~1 fold (*p* = 0.008, *q* = 0.089). Galectin-5 was only found in rat and not in any other species. Kidney IM expresses galectin-5, but its expression level is not changed in diabetes.

**Table 5:** **Summary of galectin genes from kidney IM by RNA-seq analysis**.

**Gene ID**	**Gene name**	**Average counts**		***p*-value**	**FRD**
		**Ctrl**	**STZ**		
Lgals1	Galectin-1 (Gal-1)	732.9	808.1	0.738	0.935
Lgals2	Galectin-2 (Gal-2)	NA	NA		
Lgals3	Galectin-3 (Gal-3)	117.6	209.4	0.008	0.089
Lgals4	Galectin-4 (Gal-4)	NA	NA		
Lgals5	Galectin-5 (Gal-5)	6.3	6.6	0.959	0.999
Lgals7	Galectin-7 (Gal-7)	NA	NA		
Lgals8	Galectin-8 (Gal-8)	NA	NA		
Lgals9	Galectin-9 (Gal-9)	848.4	434.2	0.001	0.019
Lgals12	Galectin-12 (Gal-12)	NA	NA		

### Validation of RNA-seq results by real-time PCR

**Figure 3:**
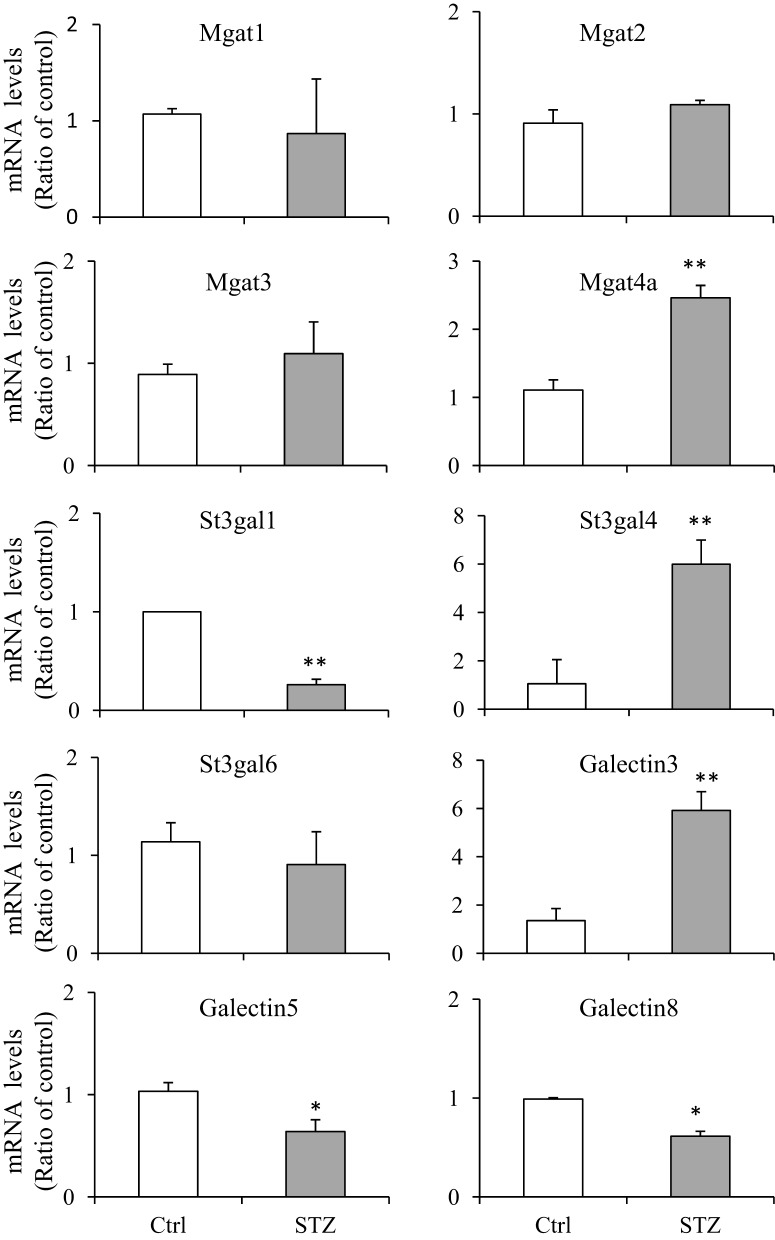
**Quantitative real-time PCR assays**. RNAs were prepared from IM tip (*n* = 3/group) and subjected to quantitative PCR (qPCR) assays using the fluorescent dye SYBR Green. The target gene mRNA levels were normalized to GAPDH. Relative mRNA levels of the target gene in STZ rats were compared to the control rats, where the expression was set to 1.0 (*n* = 3; values represent mean ± SD). A paired Student's *t*-test was used to assess statistically significant differences (compared to control ^*^P < 0.05; ^**^P < 0.01; NS, no significance).

In order to verify those glycosylation related genes identified by RNA-seq, we performed real-time quantitative PCR (qPCR). We chose 10 genes and compared their mRNA levels by qPCR. The primers, prior to using for real-time PCR, were confirmed by regular PCR as amplifying the appropriate bands and DNA gel electrophoresis showing a single expected size band (Supplemental Figure S2). Very similar patterns of gene expression were observed between the RNA-seq and qPCR analysis, however some differences were observed (Figure 3). Magt4a was found increased by qPCR but not by RNA-Seq.

## Discussion

In the current study, we show an important finding that under a diabetic condition, UT-A1 protein glycan structure is dramatically changed. UT-A1 glycan undergoes increased sialylation, fucosylation, and glycan branching. This is consistent with a recent work done by Ravidá et al. (2015) showing increased glycosylation modification of glycoproteins by terminal glucose/N-acetylglucosamine (Glc/GlcNAc), galactose/N-acetylgalactosamine (Gal/GalNAc), and fucose during disease progression of STZ-induced diabetic rat kidney cortex. Since kidney IMCD has enhanced urea permeability in diabetes (Kim et al., 2003; Pech et al., 2005), which prevents glucosuria-induced water loss, we proposed that glycosylation modification of UT-A1 by sialylation, fucosylation and branching may play an important role in regulating kidney IMCD urea reabsorption, particularly in diabetes. Although the role of glycosylation in regulating glycoprotein function has been appreciated for decades, the question of how glycosylation affects transporter activity remains unknown. Our findings indicate that when studying the regulatory function of protein glycosylation, we should go deeper to investigate how changes in the sugar component of the glycan structure may affect the glycoprotein function. Deciphering the code of each different oligosaccharide in the glycan chain may unravel how glycosylation modulates glycoprotein function.

Glycosylation is thought to be the most complex post translational modification because of the large number of enzymatic steps involved in glycan biosynthesis, glycan extension, modification, recognition, and catabolism (Nairn et al., 2008). Different forms of glycosylation are mainly due to the different processing after α-mannosidase digestion by the sequential action of specific glycosyltransferases, which generates the diverse glycoproteins. These diversities include distinct glycan composition (the types of sugars that are linked to a particular protein), glycan structure (branched or unbranched chains) and glycan length (short- or long-chain oligosaccharides). Therefore, any changes of glycan structure, length or sugar composition might affect glycoprotein functions.

Diabetes causes an increased UT-A1 protein abundance that has been recognized for years (Kim et al., 2003). The finding in this study that diabetes also causes UT-A1 glycan structure change prompts us to go to the deeper questions of how the glycan structure alteration affects UT-A1 protein function. As glycosylation is a complex process with more than 200 enzymes in 40 families (Nairn et al., 2008), to study the UT-A1 glycosylation-related genes, we employed an RNA-Seq assay. Glycan sialylation, fucosylation and branching are the three most common and important maturation processes for N-glycosylation, and these processes all occur in the Golgi complex. In this study, we are particularly interested in those enzymes involving UT-A1 glycan sialylation, fucosylation and branching.

Mgat enzymes are essential for the synthesis of hybrid and complex N-glycans, and are involved in N-glycan branching (Nishikawa et al., 1992; Lau et al., 2007; Zhao et al., 2008). Mgat3 catalyzes the addition of GlcNAc in beta 1-4 linkage to the beta-linked mannose of the tri-mannosyl core of N-linked sugar chains (Lau et al., 2007; Xu et al., 2012). The Mgat5 enzyme catalyzes tetra-antennary β1, 6GlcNAc-branched and is preferentially elongated with poly N-acetyllactosamine (Hirabayashi et al., 2002; Lau et al., 2007; Zhao et al., 2008). Mgat1, Mgat2, and Mgat4a are expressed in the kidney IM, the specific portion where UT-A1 is located. Under diabetic conditions, UT-A1 pulled down by PHA-L exhibits greater glycan branching (Figure 1). But RNA-seq analysis shows that Mgat gene expression does not increase in diabetes. We presume that UT-A1 glycan branching is not regulated at the transcriptional level but at the protein level or by increased enzyme activity. It should be noted that gene expression patterns do not necessarily correlate with protein levels (Fessler et al., 2002; Sharif et al., 2007). Alternatively, UT-A1 branching may be determined by substrate concentration. Indeed, in diabetes, the hexosamine pathway is elevated and hexosamine secretion in urine is high (Fushimi et al., 1974). The increased blood glucose and hexosamine concentrations could change UT-A1 glycosylation and lead to increased UT-A1 glycan branching. However, our qPCR data show that there is an increase of Mgat4a in diabetes, which could, together with other Mgat enzymes, promote UT-A1 glycan branching. Future studies are required to address this issue.

Glycoprotein sialylation is enhanced by sialyltransferases and reduced by neuraminidases (also called sialidases). We recently reported that sialylation increases UT-A1 urea transport activity (Li et al., 2014). In diabetes, UT-A1 glycan has increased sialylation pulled down by SNA (Figure 1), suggesting that the sialylation modification may have critical regulatory roles in UT-A1 bioactivity. There are three types of sialyltransferases which mediate α2,3, α2,6, and α2,8 sialic acid linkages, respectively. Our data show that kidney IM does not express α2,8 sialyltransferases. The α2,8 sialyltransferases St8sia2 (STX) and St8sia4 (PST) have been found to be highly expressed in brain (Rieger et al., 2008). RNA-seq analysis suggested that kidney UT-A1 sialylation may be mediated by α2,3 and α2,6 sialyltransferases. St3gal6 is the most highly expressed α2,3 sialyltransferases in kidney IM; however, St3gal4 is the most significantly changed α2,3 sialyltransferases in diabetes, and may be responsible for the increased UT-A1 sialylation.

The extent of glycoprotein sialylation can be negatively regulated by sialidases, which catalyze the hydrolysis of terminal sialic acid residues (Monti et al., 2002). At least four mammalian sialidase homologs have been described in the human genome (Neu1, Neu2, Neu3, Neu4). Igdoura et al. reported that Neu1 is highly detected in kidney (Igdoura et al., 1998). Consistent with the literature, we show in this study that kidney IM expresses Neu1. Three other neuramidase forms were undetectable in kidney. Based on the RNA-seq results, kidney UT-A1 sialylation might be mediated by sialyltransferase St3gal4 and/or St3gal6 and contracted by sialidase Neu1. However, direct evidence will be required to verify this in the future.

There are two major types of glycoprotein fucosylation enzymes, alpha (1,3) fucosyltrans-ferase and alpha (1,6) fucosyltransferase. Enhanced fucosylation levels of glycoproteins have been previously observed in both human and experimental diabetes (Poland et al., 2001; Itoh et al., 2007). Fut8 is the only member of the alpha (1,6) fucosyltransferase family and is a membrane-bound protein in the trans cisternae of the Golgi. Fut8 catalyzes the addition of fucose in the alpha 1,6 linkage to the first GlcNAc residue and form core fucosylation (Ferrara et al., 2011). Interestingly, Nairn et al reported that glycan structures containing terminal fucose residues are highly abundant in kidney (37%) and brain (19%), but have extremely low abundance in liver and testis (< 4%) (Nairn et al., 2008). It is of interest that kidney IM only expresses Fut8 but no other fucosyltransferase. We tried to clone all fucosyltransferase genes from kidney previously, but only obtained Fut8. Our RNA-Seq study confirmed that kidney IM actually only has Fut8. Therefore, we propose that kidney UT-A1 fucosylation is the core fucosylation which is pulled down by lectin AAL (Figure 1) and is most likely mediated by Fut8.

By binding glycan and forming a galectin-glycan lattice, galectin proteins play many important roles in regulating glycoprotein functions, such as stabilizing glycoproteins at the cell surface, preventing endocytosis and increasing protein stability (Rabinovich et al., 2007; Garner and Baum, 2008). Each galectin has different biological roles and recognizes different glycan structures (Stowell et al., 2008). Glycan changes often affect galectin and glycoprotein bindings. In Figure 2, we show galectin 8 does not bind to UT-A1 under normal conditions. However, during diabetes UT-A1 glycan is changed (Figure 1) and galectin-8 could bind to UT-A1 (Figure 2). Multiple galectin proteins are found in kidney IM. UT-A1 can bind to multiple galectins particularly in diabetic conditions. This suggests that UT-A1 could be regulated by multiple galectin proteins and different galectin binding may regulate UT-A1 in different aspects. We therefore propose that the formation of galectin-glycan lattices of UT-A1 binding to galectin at the cell surface leads to accumulation of functional UT-A1 on the plasma membrane and therefore increases urea transport activity.

In summary, diabetes has increased urea permeability mediated by urea transporter UT-A1 in kidney IMCD. This is consistent with increased UT-A1 protein abundance, in particular, the highly glycosylated 117 kDa form. In the current study, we show UT-A1 glycan undergoes increased sialylation, fucosylation and branching in a diabetic model. This suggests that modulation of glycan composition or structure could alter UT-A1 function. By employing RNA-seq technology, we profiled glycosylation related gene expression in kidney IM under diabetic conditions and identified some crucial genes that may mediate UT-A1 glycosylation alteration. It is important to note that in our experiments the mapped reads were relatively low. Some low expressed genes might be missed in RNA-Seq, such as Mgat3 or St3gal1 which were identified by PCR. In addition to real-time PCR, other experimental methods such as Northern blot and Western blot should be employed to provide more detailed information about these potentially important genes and gene products, as well as using immunohistochemistry to investigate their co-localization with UT-A1 and siRNA knockdown to verify their functional link. Future studies to characterize those glycosylation related genes will undoubtedly provide new insights into the understanding of how glycosylation alters UT-A1-mediated urea transport in diabetes.

## Data deposition

The RNA-seq data have been deposited in GEO with the accession number GSE69548.

### Conflict of interest statement

The authors declare that the research was conducted in the absence of any commercial or financial relationships that could be construed as a potential conflict of interest.

## Supplementary material

Supplemental Table S1 **Summary of RNA-Seq coverage data**.

Supplemental Table S2 **Upregulated network list**.

Supplemental Table S3 **Downregulated network list**.

Supplemental Table S4 **Primer sequences for qPCR**.

Supplemental Figure S1 **Volcano plot of the fold change of transcripts in control and STZ rat IM**. The x-axis is log_2_ fold change and the y-axis is -log_10_ *p*-value.

Supplemental Figure S2 **RT-PCR analysis of the primers used for real-time PCR**. Complementary DNA was synthesized from total kidney IM RNA. PCR was performed using 2-step Clontech Advantage 2 PCR kit. The PCR products were run on a 1% agarose gel (M: DNA marker).

## Abbreviations

IM: inner medulla
IMCD: inner medullary collecting duct
STZ: streptozotocin
CRD: carbohydrate recognition domain
qRT-PCR: quantitative real time PCR
Mgat: GlcNActransferase
St3gal: ST3 β-galactoside α-2,3-sialyltransferase
St6galnac: ST6 (α-N-acetyl-neuraminyl-2,3-β-galactosyl-1,3)-N-acetylgalactosaminide α-2,6-sialyltransferase
St8sia: ST8 α-N-acetyl-neuraminide α-2,8-sialyltransferase
Neu: neuraminidases
Fut: fucosyltransferase.
