# Supplementary material for: RNA-seq analysis of glycosylation related gene expression in STZ-induced diabetic rat kidney inner medulla
Source: Front Physiol. 2015 Oct 1;6:274. doi: 10.3389/fphys.2015.00274 (PMC4590316; doi:10.3389/fphys.2015.00274)

Supplemental Table S1

Table S1. Summary of RNA-Seq coverage data

Group	Sample ID	Yield (Mbases)	# Fragments	Mapped fragments
Ctrl	Ot3046	2395.370600	11976853	6,987,341 (58.34%)
	Ot3047	2820.203000	14101015	9,533,574 (67.61%)
	Ot3048	2591.304000	12956520	7,640,811 (58.97%)
STZ	Ot3052	2863.229800	14316149	7,610,211 (53.16%)
	Ot3053	2918.117000	14590585	8,467,302 (58.03%)
	Ot3054	3238.085400	16190427	8,900,241 (54.97%)

Supplemental Table S2: Upregulate Network List

ID	Molecules in Network	Score	Focus Mole	Top Functions
1	Angiotensin II receptor type 1, ↑**BMP3**, ↑**CCBP2**, ↑**CDC42EP5**, ↑**CIRBP**, ↑**CRHR1**, ↑**DGKI**, ERK1/2, ↑**GFRA2**, ↑**GHRH (includes EG:14601)**, ↑**GHSR**, ↑**GLP1R**, ↑**GNAZ**, ↑**GNRH1**, ↑**HSD17B3**, Insulin, ↑**LHCGR**, ↑**NEU1**, ↑**NPY**, ↑**NPY2R**, Nr1h, ↑**OPRK1**, ↑**Prl3d1 (includes others)***, Proinsulin, ↑**PTGIS**, ↑**PTPRVP**, ↑**RETN**, ↑**RGS17**, Rxr, ↑**SERPINA12**, ↑**SH3BP2**, ↑**SULF1**, ↑**UCN2**, ↑**UTS2**, ↑**UTS2R**	32	29	Cell Signaling, Molecular Transport, Nucleic Acid Metabolism
2	Alpha catenin, ↑**CAMK4**, Cg, ↑**CGA**, ↑**CNGA3**, Collagen type I, ↑**CRX**, ↑**CYP17A1**, ↑**CYP2D6**, ↑**CYP4B1**, ↑**DDR1**, ↑**DLX3**, ↑**DMP1**, ↑**DSPP**, ↑**EDN3**, ↑**GABPB1**, ↑**GSTM1**, ↑**IHH**, ITPR, ↑**JPH1**, LDL, ↑**NFATC4**, ↑**OXT**, ↑**OXTR**, ↑**PDE6A**, ↑**PDE6C**, PP2A, ↑**PPARG**, ↑**PRKG2**, ↑**RARRES2**, ↑**SERPINA1**, ↑**SFRP4**, ↑**SULT2A1***, ↑**TLL1**, ↑**TRDN**	32	29	Tissue Morphology, Gastrointestinal Disease, Neurological Disease
3	↑**5430435G22Rik**, ↑**ABCD3**, ↑**ACER2**, ↑**ALK (includes EG:11682)**, ↑**BCL11A**, ↑**BPI**, caspase, ↑**CYP3A4**, ↑**CYP3A5**, ↑**DCC**, ↑**DMBT1**, ↑**FABP6**, ↑**GZMK**, ↑**HIF3A**, ↑**IDO1**, IFN Beta, IFN TYPE 1, ↑**INSRR**, ↑**IRF4**, ↑**IRF8**, ↑**LMO4**, ↑**LY96 (includes EG:17087)**, ↑**MESP2**, MHC Class II (complex), ↑**mir-181***, NFkB (complex), ↑**NTN1**, ↑**NTN3**, ↑**NTRK3**, PLC gamma, ↑**PPP1R16B**, ↑**PTPRZ1**, ↑**SFTPA1**, ↑**SPIB**, Tlr	30	28	Cellular Growth and Proliferation, Nervous System Development and Function, Cellular Development
4	Alp, ↑**BGLAP**, ↑**CENPA**, Collagen Alpha1, ↑**CORT**, ↑**FBLN1**, ↑**GFAP**, ↑**HAS1**, ↑**HSD3B1***, ↑**HTR2B**, IFN alpha/beta, ↑**IGFBP2**, ↑**LHB**, ↑**Ly6a (includes others)***, ↑**MMP10**, ↑**MPZ**, ↑**MSMB**, ↑**MXD3**, ↑**NOG**, ↑**NOTCH1**, ↑**PAPPA**, ↑**PORCN**, ↑**PTPRK**, pyruvate kinase, ↑**SGCA**, Smad, Smad2/3, ↑**ST8SIA2**, ↑**TAGLN**, Tgf beta, ↑**TGFB1**, ↑**TGFBI**, ↑**VNN1**, ↑**WISP1**, ↑**WNT11**	30	28	Cellular Development, Skeletal and Muscular System Development and Function, Cellular Movement
5	↑**BHLHE23**, CD14, CDH2, CDK5, ↑**CER1**, CTNNB1, ↑**FAM213A**, ↑**FGD2**, GATA2, GNB2L1, HSPA8, IGF2, ↑**IRF4**, ↑**ITGA10**, ITGB1, estrogen receptor, ↑**FLNC**, FSH, ↑**GP9**, Hdac, Histone h3, Lh, ↑**MEF2D**, ↑**MSC**, ↑**NOTCH4**, ↑**PCYT1B**, PDGF BB, ↑**Peg12**, ↑**PHKA2**, ↑**PPY**, ↑**RAB5A**, ↑**ROBO2**, ↑**STS**, ↑**TFAP2C**, ↑**TNNI1**, ↑**UGT2B17***, Vegf, ↑**VEGFA**	30	28	Developmental Disorder, Skeletal and Muscular Disorders, Endocrine System Development and Function
6	↑**AGAP2**, Akt, Alpha Actinin, Ap1, ↑**ASAH2**, ↑**ASGR1**, ↑**Calm1 (includes others)***, Calmodulin, CaMKII, ↑**Ctf2**, ↑**DRD3**, F Actin, ↑**HCLS1**, Hsp70, Hsp90, ↑**HTR1A**, ↑**ITPKA**, ↑**KCNQ1**, ↑**LGI3**, ↑**LINGO1**, ↑**LRRC7**, ↑**MST1R**, ↑**NPAS2**, ↑**PACRG**, ↑**PDCD6IP**, ↑**PDLIM2**, ↑**PGLYRP1**, Pka, ↑**PKIA**, ↑**RAB3A**, ↑**SCGB3A1**, ↑**SLC6A2**, ↑**SYN1**, ↑**SYN2**, ↑**SYT9**	27	26	Cell-To-Cell Signaling and Interaction, Drug Metabolism, Molecular Transport
7	↑**ACTC1**, ↑**ASCL1**, ↑**BMPER**, CD3, ↑**CD52**, ↑**CDKN2A**, Creb, ↑**CREM (includes EG:12916)**, Ctbp, Cyclin A, ↑**DBH**, ↑**ELF4**, ↑**FAM129A**, ↑**GPT2 (includes EG:108682)**, ↑**GRAP2**, ↑**HAND2**, Histone h4, ↑**LAG3**, ↑**LPIN2**, ↑**LRRN3**, Mapk, ↑**NHLH2**, ↑**PTGER4**, Ras, ↑**SATB1**, ↑**SIK1**, Smad1/5/8, ↑**SOSTDC1**, ↑**SYNPO**, TCR, ↑**TNFRSF8**, ↑**TPPP2**, ↑**TRPV6**, ↑**VPREB1***, ↑**VPS37B**	27	26	Cell Cycle, Hematological System Development and Function, Hematopoiesis
8	↑**CCL17**, ↑**CCL20**, ↑**CCL3L1/CCL3L3**, ↑**CD5**, Ck2, ↑**CLCN1**, Cpla2, ↑**CSF3R**, ↑**DLGAP2**, ↑**DLX1**, Fc gamma receptor, Fcer1, ↑**FES**, ↑**FMO1 (includes EG:14261)**, ↑**GRIN2B**, IL1, IL23, ↑**IL33**, ↑**IL17A**, Jnk, ↑**Klra4 (includes others)***, ↑**LIN7A**, NADPH oxidase, ↑**NCF2**, P38 MAPK, ↑**PLP1 (includes EG:18823)**, ↑**POSTN**, ↑**SLC12A2**, ↑**SLC30A3**, ↑**SLC4A2**, ↑**SRSF9**, SYK/ZAP, ↑**TNFRSF17**, ↑**TNFSF13**, ↑**WNT3**	25	25	Hematological System Development and Function, Inflammatory Response, Tissue Morphology
9	↑**A2M**, ↑**ABCB4**, ↑**ABCG8**, AMPK, amylase, ↑**APOA4**, ↑**APOA5**, ↑**APOBEC1**, ↑**ARR3 (includes EG:170735)**, ↑**BHLHA15**, ↑**CDK5R1**, ↑**CHRNA9**, Collagen(s), ↑**CPA1 (includes EG:109697)**, creatine kinase, Cytochrome c, ERK, ↑**FOSL1**, GOT, HDL, HDL-cholesterol, ↑**HFE**, ↑**HTR1B**, LDL-cholesterol, ↑**LPL**, ↑**MYL2**, ↑**MYL7**, ↑**PTGES**, ↑**RXRG**, ↑**SDC1 (includes EG:20969)**, Secretase gamma, ↑**SLC22A7**, ↑**SLCO1B3**, ↑**UCP3**, VLDL-cholesterol	22	23	Lipid Metabolism, Molecular Transport, Small Molecule Biochemistry
10	↑**AADAC**, ↑**ADH4 (includes EG:127)**, ↑**AGR2**, ↑**AICDA**, C5, ↑**COX6A2**, CYLD, ↑**F11**, ↑**FBP2**, ↑**FGF8**, ↑**FOXA2**, FOXA3, ↑**FXYD3**, ↓GCG, ↑**GCNT3**, ↑**GOLT1A**, ↑**HNF1A**, ↑**HSD17B2**, ↓IGFBP1, IgG2a, IgG2b, ↑**ITIH4**, LTA, ↑**MFI2**, ↑**MYL9**, ↑**PDK1**, ↑**PFKL**, PITX2, POU2AF1, PRLR, RNA polymerase II, ↑**Sult1d1**, ↑**TAT**, ↑**TNNT1**, ↑**UGT2B10**	22	23	Humoral Immune Response, Protein Synthesis, Cancer
11	14-3-3, ↑**AICDA**, ↑**ATXN1**, BCR (complex), ↑**CA3**, ↑**CD80 (includes EG:12519)**, ↑**CSN2**, ↑**CTSE**, ↑**CXCL1**, ↑**FGF21**, Fibrinogen, Focal adhesion kinase, Gm-csf, Growth hormone, ↑**H19**, ↑**HAVCR1**, ↑**HOXC4**, Iga, Ige, IgG1, Igg3, Igm, ↑**Il3**, ↑**IL5RA**, ↑**KNG1**, PI3K (complex), ↑**PIGR**, ↑**PIM1 (includes EG:18712)**, ↑**PTGDR2**, Rac, ↑**SELP**, ↑**SLC1A6**, ↑**ST3GAL4**, STAT5a/b, ↑**VAV3**	19	21	Humoral Immune Response, Protein Synthesis, Tissue Morphology
12	↑**1700009N14Rik**, ↑**ARMC12**, BCL6, BMP4, ↑**C11orf96**, ↑**C1orf96**, ↑**CRX**, ↑**CYP17A1**, FIGLA, GLI1, GNB1, GNB2L1, ↑**GREM2**, ↑**GUCA1B**, ↑**GUCA2B**, ↓GUCY2F, HDAC2, ID2, ↑**IMPDH1**, ↑**IRGC**, ↑**KLHL10**, ↑**LPCAT1**, LRCH4, MEF2C, NRL, ↑**PDE6A**, PDE6B, PDE6G, RHO (includes EG:212541), ↑**RLBP1**, ↑**RLIM**, SHH, SMAD4, SP3, ↑**TULP1**	13	17	Hereditary Disorder, Ophthalmic Disease, Neurological Disease
13	ADM, ALOX5, ↑**ALOX15B**, ↑**ANKRD37**, ↑**ARSI**, B2M, ↑**Calcb**, ↑**CCL17**, ↑**CCL20**, CHRNA7, ↑**CLEC4D**, ↑**CUL4B**, CXCL6, ↑**CYP26B1**, ↑**D4S234E**, HLA-C, ID1, IFN Beta, IFNAR1, IL28RA, ↓ISG15, ITGAV, LTA, MHC Class II (complex), NEDD9, ↑**PLA2G3**, ↑**RIC3**, ↑**SLAMF7**, ↑**SLC28A1**, SOD2, THBS1, TNF, ↑**TREM2**, VAV1, ↑**Vmn1r49 (includes others)***	12	16	Immunological Disease, Infectious Disease, Dermatological Diseases and Conditions
14	ACOX1, ALOX5, AMPK, APOA1, ↑**BRS3**, ↑**CEL**, ↑**CHRNB2**, CNR1, CNTF, ↑**CPS1**, CRH, ↑**CRHBP**, CRHR2, ↑**CYP1A2**, ↑**CYP2F1**, ↑**DAGLA**, ↑**ERN2**, G6PC, ICAM1, IL1R1, ↑**ITLN1**, ↑**LEAP2**, ↓LEP, ↓MT1E, ↑**NPY**, NR1I3, PCK1 (includes EG:18534), ↑**PON3**, ↑**PRLH**, ↑**PTGER1**, REST, ↑**SELP**, ↓TNFRSF1B, UCP2, ZBTB20	12	16	Digestive System Development and Function, Metabolic Disease, Lipid Metabolism
15	↑**ACTA1**, ↑**AICDA**, ↑**AMY2A**, ↑**BCAN**, BNIP3L, ↑**CCDC164**, CCR2, CDKN2B, ↑**CRISP3**, CSF2RB, ↓E2F1, FGFR2, IL5, ↑**IL5RA**, ↓INHA, ↑**IRF4**, ↓KLF4, ↓KLF10, let-7a-5p (and other miRNAs w/seed GAGGUAG), ↑**LRRN1**, ↑**mir-223**, ↓MT1E, ↑**MYBPH**, MYC, ↑**MYO7A**, ↑**PDIA2**, ↑**PGC**, Rb, RBBP4, SKP2 (includes EG:27401), SMARCA2, SMARCA4, ↑**THOP1**, ↑**TNNC2**, XBP1 (includes EG:140614)	12	16	Hematological System Development and Function, Tissue Morphology, Cellular Development
16	↑**BHLHE23**, CD14, CDH2, CDK5, ↑**CER1**, CTNNB1, ↑**FAM213A**, ↑**FGD2**, GATA2, GNB2L1, HSPA8, IGF2, ↑**IRF4**, ↑**ITGA10**, ITGB1, ↑**KCNIP4**, ↑**KLK1***, LEF1, ↑**LIM2**, miR-483-3p (miRNAs w/seed CACUCCU), MITF, ↓MPO, ↑**MYF5**, ↑**NEUROG1**, NR2E1, PDGFRB, ↑**SPAG11B**, ↓SPI1 (includes EG:20375), TDGF1, ↑**TNIK**, ↑**TUBA3C/TUBA3D***, USF1, ↑**WASL**, ↓WNT1, WNT3A	11	15	Embryonic Development, Organismal Development, Cellular Development
17	↑**ADAMTS2**, AHR, ↑**ALDH1B1**, ↑**APOC4**, ARNT, ↑**CCL20**, CCND3, CDH2, ↓CEBPA, ↑**CSF3R**, CYP19A1, ↑**CYP1A2**, ↓CYP1A1 (includes EG:13076), ↑**FCAR**, ↑**GALNT13**, ↑**HLA-DOB**, ↑**HPGD**, Hsp90, ID2, IL21, IL27, ↑**KIF26B**, KIT, ↑**LRFN5**, ↓MT1E, NQO1, ↓NR1I2, ↑**NRG4**, ↑**OLFML1**, PAX7, PCK1 (includes EG:18534), REL, ↑**RNF144A**, TGFB2, WNT3A	11	15	Digestive System Development and Function, Hepatic System Development and Function, Organ Morphology
18	↑**AKAP6**, ↑**ARNTL2**, ↑**BCL11B**, ↑**CAPN11**, ↑**CCL20**, CD14, ↑**CD300LB**, CTCF (includes EG:10664), CYBB, F13A1, ↑**FCRL6**, Gm-csf, HLA-DRB1, ↑**HTRA4**, IL2, IL13, IL1R1, ↓IL1RL1, IL23A, ↑**IL36RN**, ↑**IRF4**, ↑**LSM11**, LTA, NFATC2, ↑**NRIP3**, ↑**PCDHAC1**, ↑**PCDHAC2**, PDE4D, POU2AF1, TBX21, ↓TERT, THBS1, ↓TNFRSF1B, ↑**TPSG1**, TREM1	11	15	Cell-To-Cell Signaling and Interaction, Hematological System Development and Function, Inflammatory Response
19	App, BCL2L1, ↑**C1QTNF7**, CASP9, CCL4, ↑**CCL3L1/CCL3L3**, ↑**CKB**, CRH, CRHR2, ERN1, ↑**Grifin**, ↑**HBZ**, HES1 (includes EG:15205), IKZF1, LATS1, ↑**LHX8**, ↑**MOB1A**, ↑**MORC1**, ↑**NLRP10**, ↑**NOTCH1**, NR1H4, NR3C1, PAK1, POMC, PSEN1, PSEN2, ↑**PSTPIP1**, SCARB1, ↑**SLC1A7**, SNCA, ↑**SNCB**, SYP, ↑**SYT13**, ↑**TBX19**, TH	11	15	Behavior, Embryonic Development, Nervous System Development and Function
20	APC, ↑**BRSK1**, CACNA1A, CACNA1B, ↑**CAMKV**, CDH2, ↑**CNNM2**, ↓CXCL2, CXCL3, ↓DPP4, ↑**DPP6**, ↑**EXTL1**, ↑**FAP**, ↓FGF2, ↑**GIP**, GNB1, ↑**KCNC3**, ↑**LCT**, ↑**LPL**, ↓MMP3, NPPA, ↑**PCLO**, PRKCA, Proinsulin, ↑**RASL10B**, RHOA, RTN4 (includes EG:57142), ↓SLC2A4, ↑**SLC6A5**, SPDEF, ↑**SPEG**, STX1A, TJP1, ↑**UNC13A**, Vegf	11	15	Cell-To-Cell Signaling and Interaction, Molecular Transport, Small Molecule Biochemistry
21	↑**ACTA1**, ACTA2, BMP4, ↑**CACNA1D**, ↑**CCDC33**, ↑**DBX1**, ELK1, EPS15, ↑**FCHO1**, ↑**FUT9**, LHX1, ↑**LMOD1**, MEF2C, MYOCD, MYOD1, NPPA, ↑**NUMBL**, ↑**OR2AK2***, PAX6, POU4F2, POU5F1, ↑**PPP1R12B**, PRDM5, PRKAR1A, ↑**RERGL**, RUNX1, RUNX2, ↑**SCN5A**, SHH, SOX2, SRF, ↑**TEAD1**, ↑**TMEM229A**, ↑**UBL7**, UPF2	11	15	Cellular Development, Gene Expression, Embryonic Development
22	↑**AADAC**, ATF4, ↑**CPN2**, ↑**DDIT4**, EIF4EBP1, FOLR1, ↑**GIMAP1**, ↑**GLDN**, ↑**JAKMIP2**, ↑**JPH2**, LPPR4, miR-200a-3p (and other miRNAs w/seed AACACUG), ↑**OLAH**, PKD1, PKD2 (includes EG:18764), POU2F1, ↑**PPM1E**, PPRC1, PRKCSH, PRLR, ↑**PRODH2**, ↑**SLC13A2**, ↑**TRIM67**, VIM, ↑**Wfdc3**	11	13	Reproductive System Disease, Organ Morphology, Renal Dilation
23	26s Proteasome, ↑**ACRV1**, ↑**ADAM23**, AVP, BCL2L11, ↑**CA1**, ↑**CIT**, CLOCK, COL18A1, CREB1, CREBBP, ↑**Csn1s2b**, DLG4, ELK1, ↑**GABRD**, ↑**GPR88**, ↑**GRIK2**, GSK3B, HTT, IGF2, IGFBP5, ↑**LDHC**, ↑**LOXHD1**, ↓MMP3, ↑**MYL2**, ↓NFIL3, PDGFRB, ↓POR, ↑**PROK2**, PTEN, ↑**RAP2B**, SUZ12, ↑**TBX6**, TH, YBX2	10	14	Neurological Disease, Psychological Disorders, Behavior
24	ADRB3, ADRBK1, ↑**AKNA**, CNTF, ↑**CPLX1**, CRH, ↑**CYP2C8**, ↑**DBH**, ↑**DPYD**, DRD1, DRD2, ↑**EPN1**, F2, F2R, ↑**FGF14**, FOS, GAL, GH1, Gh, GHR, ↑**GHRH (includes EG:14601)**, ↓IGFBP1, ↑**LOC259246 (includes others)***, ↑**NAT8L**, ↑**NCS1**, PCSK1, ↑**PKIB**, ↑**RIMS2**, SBDS, SLC6A3, SST, SSTR2, TRH, ↑**TRHR**, ZBTB20	10	14	Nutritional Disease, Endocrine System Development and Function, Nervous System Development and Function
25	CASP7, CDK6, CDKN1B, ↑**CRYGB**, ↑**CRYGD**, ↑**DAZAP1**, ↑**DBC1**, DCN, DKK1, FOXM1, G6PC, ↑**GJB6**, ↑**HCN4 (includes EG:10021)**, HIPK2, ↑**KCNN4**, ↑**KRT1**, MAF, ↑**NELL2**, ↑**ODZ4**, PARP1, PARP, PDE6B, ↑**PIK3C2B**, PITX2, POU4F2, RB1, RBBP4, SIRT1, SKP2 (includes EG:27401), ↑**SLURP1**, Sos, ↑**TBX2**, THRB, TIMP2, ↑**ZIC2**	10	14	Cell Morphology, Embryonic Development, Organ Development

Supplemental table S3: Downregulated Network List

ID	Molecules in Network	Score	Focus Mole	Top Functions
1	**BTNL2,** Calbindin, **CD55, CD200R1, CLDN2, CMKLR1, COL17A1, CPB2, CPEB1, CRMP1, FGB, FGG, GPNMB, GPR132, HLA-DMA,** Igfbp, **IL6, IL20, IL9R, LALBA, LPAR3,** lymphotoxin-alpha1-beta2, **MEP1A, NEU3, NLRP12, NMU, P2RY6, PBK, PDE5A, Prl3b1, Prl3d1 (includes others)*,** Ras homolog, **S1PR2, SLC10A1, SLC22A3**	31	31	Reproductive System Disease, Cell Cycle, Hematological System Development and Function
2	**ADRA1A, ADRA1D, ADRB1, AQP5, BTC, CRYGC, CSRP2, DEF6, EMCN, ENC1,** ERK1/2, **FGF7, GRP, HSF4, ITGA7, KLRD1,** L-type Calcium Channel, **MFGE8, MMP7, NDST1, NFIX, NPPC, PDE6H, PROCR, PRR7,** Ras, **RASGRF2, RASGRP3,** Rsk, **SIT1 (includes EG:27240), SPZ1, SSTR3, SULT1E1, THPO, TRAT1**	31	31	Cardiovascular Disease, Heart Failure, Developmental Disorder
3	**Adam3, AFM, AIRE, BMP10, CALB2, CD9, CD46, CD84, COL13A1,** estrogen receptor, **FABP1, FGF1, FNDC1,** G-protein beta, **GNAO1, GPRC5A,** Hdac, **HLA-E,** IgG, **IGSF8, JPH3, KRT13, MAB21L1, MMP15, MTNR1A, MTNR1B, Ngp, OSR1, PCDH8, PNLIPRP1, SERPINB7, SMAD6, TFF3, TGFB3, ZDHHC2**	31	31	Cell-To-Cell Signaling and Interaction, Reproductive System Development and Function, Tissue Development
4	**ACTBL2,** APC (complex), arginase, **BUB1 (includes EG:100307076), CALCR,** Cbp/p300, **CCL24, CCNB1, CCR3, CCRL2, CDC20 (includes EG:107995), CKAP2, CYSLTR2, F9,** Filamin, **GATA1, GNA15, GP5, GP1BA, HAS3, HIST1H2AB/HIST1H2AE, HSD3B1*, IFI27L2, IFIT3,** IFN alpha/beta, **IL20RB, IL4 (includes EG:16189), IRGM,** Angiotensin II receptor type 1, C/ebp, **CCL28,** Collagen type III, **Csn1s2a,** Cyp2b, **DSG3,** Fc gamma receptor, **FCER1A,**	29	30	Cardiovascular System Development and Function, Cell-To-Cell Signaling and Interaction, Respiratory System Development and Function
5	**ALDOB, ALOX12B,** ALT, **AVPR1B, BCMO1, BHMT, CIDEB, CIDEC, CYP2C9, DLL3, ENPP1, FOXG1, GABRB3, GBX2,** Glycogen synthase, **HES5, HRH3, HTR2C, LEP, LGALS12, MECP2, MSX1, MT1E, NES,** Notch, **NPY1R, NTF4,** Proinsulin, **PTPRN2, QRFP, T, TTPA, WIF1, WNT1,** Wnt	29	30	Nutritional Disease, Connective Tissue Development and Function, Tissue Morphology
6	**CDK1, COL5A1,** Cyclin A, **DBF4 (includes EG:10926), DNTT, E2F1, E2F3,** E2f, **ESRRB,** Gamma tubulin, **GRK1, GUCA1A, GUCY2D, GUCY2F, HIST1H3A (includes others),** Histone h3, **HMGA2, mir-24*, MYLPF, MYT1, OPN1LW, PCSK1N, Pmaip1, PRR11, RACGAP1,** Rb, **S100A8, SCRT1, SPATA18, SPATS2L, STAG3, STOM, TERT, TP63, ZNF365**	29	30	Cell Morphology, Embryonic Development, Organ Development
7	**APOH, CALB1, CAMK2D, CARD6, CDH22,** Fibrin, **FOXN1, GZMA, HABP2,** IKK (complex), **IL1RL1,** IRAK, **LCA5,** Mmp, NFkB (complex), **NGFR, NLRP4, NTF3, PELI3, PKMYT1, PLG,** PP2A, **REG3A, RTKN, SERPINC1, SERPINF2, SLC2A5, Slpi (includes others)*, ST18, ST3GAL1, SYT6, TAP2, TRAF6, TYRO3, ZBTB32**	27	29	Cell Morphology, Nervous System Development and Function, Tissue Morphology
8	**ADCYAP1R1,** Alp, **ATF3, CCL7,** Cg, **COL11A2,** Collagen Alpha1, **DPT, EGF, EGR1, EMB, FAS, FBP1, FGF2, GDF10, Giot1/Zfp347*, HSD17B1, KLHDC4, LUM, MGP, NPR3 (includes EG:18162), NUAK2, Oasl2, OGN,** PARP, Pkc(s), **PLCD1, SCG2, SERPINB5, SERPINE1, SLC16A2, SLC34A1, TPO,** TSH, **WNT10B**	27	29	Cell Morphology, Cellular Assembly and Organization, Embryonic Development
9	**ACTG2, AGTR1,** Ap1, **AQP8, AQP12A/AQP12B, ARID5A, BAAT, CBS, COCH, CYP1A1 (includes EG:13076), CYP7A1, ELOVL3,** FSH, **GPR12, GSTA5,** HDL-cholesterol, **Hsd3b4 (includes others), Ifi47, IFIT1B, INHA, LAMC3,** LDL-cholesterol, Lh, **LHX9, NCOR2, NDST3, NR0B2, NR1I2, NR5A1, NR5A2, P2RY13, POR, RGS12,** RNA polymerase II, **TP53I11**	27	29	Lipid Metabolism, Molecular Transport, Small Molecule Biochemistry
10	**ALB,** BCR (complex), **CD38, CD72 (includes EG:100427076), CEBPA, CFD, CHI3L1,** Collagen type IV, **CSF3, CYP2B6*, EPX,** Fibrinogen, **FOSB, GCH1, GFPT2, HEPACAM2, HTR5A,** IgG1, Igm, **LPXN,** MAP2K1/2, Mapk, **Mcpt1*, MPO, NDUFV3, NFIL3,** PDGF BB, **PTPN22, RGS16, RGS20, SCGB3A2, SIGLEC10, SLFNL1, SPI1 (includes EG:20375), VCAN**	24	27	Cancer, Cellular Development, Tumor Morphology
11	**CCR7, DDN, DHX58, FUT8, GSTA1,** Ifn, IFN Beta, IFN TYPE 1, Ifnar, **IL25,** IL12 (complex), **IL12B, IL36B, IRF7,** Jnk, **KIRREL, KLF15, MAP2K4, MAP2K7, MAPK8IP1,** MHC Class II (complex), Mlc, **Ms4a4b (includes others), NEFH, NEFL, NEFM, NPHS1, NPHS2, TLR6,** Tlr, **TNFSF15, TNN, TRIM21, TRPC5, TRPC6**	22	26	Cell-To-Cell Signaling and Interaction, Cellular Assembly and Organization, Cellular Function and Maintenance
12	ADCY, AMPK, **CACNA1H,** calpain, **CHRNA10, DAO, DUSP4,** ERK, G protein alphai, **GALR1, GALR2, GCG, GCGR, GCLC, GHRL, GNAT3, GNRHR, GUCY1A2,** JAK, **KLF10, LTB4R, MAFF, MAP1B,** Nuclear factor 1, **P2RY12, PCSK2,** PI3K p85, PLC, **PPP2R2C, PTHLH,** Rap1, **S100G, SEMA3A, SERPINA3, SLC2A2**	20	25	Cancer, Endocrine System Disorders, Gastrointestinal Disease
13	**ADRB2, AKAP5,** Akt, Ampa Receptor, **ANGPT2, APLNR, CACNG2,** Calmodulin, **CDH13, Ces1e, CNTN2, GRIN1, GRIN3A, HPSE, HTR2A, ICAM5,** Kallikrein, **KCNN2, KCNQ3, KLK1*,** Ldh, MTORC2, Myosin, **OPRD1,** p70 S6k, **PHLPP2, PIK3R5,** Pka, PP1 protein complex group, Pp2b, **Psg16*, SEC14L2, SLC1A1, TSPAN17, VAMP1**	19	24	Organismal Injury and Abnormalities, Neurological Disease, Psychological Disorders
14	**ACACB, APOC2, BDKRB2, BMP15, C6,** C8, **C9, CCL13, Ces1d,** CPT1, **CXCL2, CYP24A1,** HDL, **ISG15, ISG20,** JINK1/2, **KLF2, MN1, MOGAT2, MX1*,** N-cor, Nr1h, PEPCK, PI3K (family), **PRELP,** PRKAA, **PTGS2,** Rxr, **SLC20A1, SLC22A1, SOAT2, TACR1, TLR5, USP18,** VLDL-cholesterol	19	24	Lipid Metabolism, Small Molecule Biochemistry, Molecular Transport
15	Angiotensin II receptor type 1, C/ebp, **CCL28,** Collagen type III, **Csn1s2a,** Cyp2b, **DSG3,** Fc gamma receptor, **FCER1A, HCRT, HRH1,** IL1, **KRT14, LBP, LBX1, MAP3K6, MAPK11, MMP3, Myh11,** Nfat (family), **OLR1,** P38 MAPK, **PLA2G7, PLN, PPP1R1A (includes EG:5502), PTAFR,** SAA, SERCA, **SLC18A3,** Sod, SYK/ZAP, **TNFRSF18, TNFRSF1B, XPNPEP3, YAP1 (includes EG:10413)**	18	23	Nervous System Development and Function, Developmental Disorder, Lipid Metabolism
16	**ACAN, CCL1, CCL2, CD274,** Cdk, chemokine, **CLEC4M, CXCL10,** Cyclin E, **ETV5, FGFBP1, GJB2, GPR31,** Gsk3, Hsp27, **HSPB3, IFI44, IFIT2,** IgG2c, **IL15 (includes EG:16168), IL7R,** Immunoglobulin, Interferon alpha, **KLF4,** Mek, **MERTK, MLANA,** MTORC1, **POLQ, PPBP, RAB23, RRM2B,** STAT5a/b, TCR, **ZBTB17**	18	23	Cell-To-Cell Signaling and Interaction, Cellular Movement, Hematological System Development and Function
17	**BLNK,** Calcineurin protein(s), **CCR6,** CD3, **CD4, CD28, CSRP3, CXCR6, CYGB, EGR2,** Fcer1, **FCGR2B,** Gm-csf, **GRASP, GRM1, HOXA1,** Ige, Igg3, IgG2a, IgG2b, **ITGAD, KMO, Mbl1, MME, MSN,** NFAT (complex), PLC gamma, **RAG1, RLN1,** Rock, Shc, **SIAE, SLC1A3, SOX10,** Tgf beta	16	22	Humoral Immune Response, Protein Synthesis, Hematological System Development and Function
18	**ABL2,** Cdc2, **CHGA,** Cofilin, **CUZD1,** F Actin, Focal adhesion kinase, **FOXE1, GAP43, Gbp2, GJD2,** GOT, **GRM7,** Growth hormone, **HMMR, IGF1, IGFALS, IGFBP1,** Insulin, Laminin, **LEPR, LIMK1, Mcpt8*, MT3, NEUROG3,** p85 (pik3r), **PAX4,** PFK, PI3K (complex), pyruvate kinase, Rac, **SLC2A4, SLC4A1,** SRC (family), **UCP1**	15	21	Nervous System Development and Function, Tissue Morphology, Carbohydrate Metabolism
19	**AMY1A (includes others), DEFB106A/DEFB106B,** DIO3, DMD, **EPHX4,** GSTP1, HDAC1, **KCNJ10, KCNK2,** KLHL4, **KRT25, KRT26,** MBD2, **Mill1,** NEIL3, **Olfr711, PRSS16,** PSEN1, PTF1A, **S100A5, SEMA3D, SERPINB12, SHISA7, SLC23A1, SUSD4,** ZNF217	12	16	Cell Morphology, Amino Acid Metabolism, Molecular Transport
20	26s Proteasome, Alpha tubulin, **ATP6V1B1, AZGP1,** C5, **C15orf59,** CASP3, **DISC1, FOXI1,** GADD45A, GADD45B, **GPR1 (includes EG:100004124), HDHD1,** Hsp70, Hsp90, **INHBE,** MAOA, MIF, **O3FAR1,** PIM1 (includes EG:18712), **PLK1,** PRDM5, PRKAA2, PRNP, PTK2, **RHAG, RHCE/RHD,** SBDS, **SLC26A4, SLC4A1, SORCS1, STK32C, STOX1,** Ubiquitin, **USP13**	11	18	Renal and Urological System Development and Function, Metabolic Disease, Renal and Urological Disease
21	**AKR1D1, ATP2B3,** BCL2A1, CACNA1A, CACNA1B, CADM1, **CADM3, CELSR3,** CLTC, **Cyp2b13/Cyp2b9,** DLG1, DMRT1, **DMRT3, DMRTA1, FETUB,** FOXL2, **GPR158,** GRM5, KIF2A, **LGI1,** MARK2, **MRGPRE,** Nrg1, **PARPBP, PNMA1, RAB3B, SAMD4A, SLC8A2,** SNAP25, Sos, STAT5A, **TAS2R13, TNMD,** YWHAG, YWHAH	11	18	Cell-To-Cell Signaling and Interaction, Cellular Assembly and Organization, Cellular Function and Maintenance
22	AR, CCND1, CD151, CDK5, CSF1R, DMRT1, **ERVFRD-1, FAM83D, FOXH1,** FSHR, **GATA1,** GJA1, GLI2, GLI3, **HIST2H2BE (includes others), HOXB13, HSD3B1*, KRT80,** LEF1, **LIN7B,** MAOA, MED1 (includes EG:19014), MTA1, NCOA1, NCOA3, **NRSN1,** PTH1R, **SIX3, SLC38A5, SLC9B2,** SPINK1, **Syna, TPPP, ZIC1, ZNF521**	10	17	Organismal Development, Reproductive System Development and Function, Tissue Development
23	AHR, CCND3, CCNE1, CD44, CDKN2A, CDKN2B, **CHRNA2, CHRNB4, CYP2S1,** DUSP1, EIF2AK3, **FMO3,** FN1 (includes EG:100005469), Hdac, Histone h4, **IL15 (includes EG:16168), ITGBL1, KCNJ8, OLR1, POLR2B,** POU4F1, **PTRF, Rdh1 (includes others), SERPINE1, SLC12A3, SLC1A4, SLC35A5,** SMAD4, **SYT12,** SYVN1, Tgf beta, TGFB2, TGFBR2, **ZBTB26,** ZFP36	10	17	Cellular Development, Cellular Growth and Proliferation, Connective Tissue Development and Function
24	**ABO*, AOC3,** ARRB2, **BMP8B,** CLU, CTTN, **CX3CR1, DPYS, DSC3,** ELANE, Gsk3, HSF1 (includes EG:15499), ICAM1, ID1, ITGAL, **LOC653486/SCGB1C1,** LTBR, MAPKAPK2, MIF, MKNK1, **NR5A2,** PIK3CG, PTX3, **Rcan2, RGD1562525 (includes others), RGS14,** SELP, SERPINA1, **SERPINB10, SLC12A1, SPSB1,** SRC (family), **TEX12, TMPRSS3,** TNF	9	16	Hematological System Development and Function, Immune Cell Trafficking, Inflammatory Response
25	**ACOXL, AGPAT5, AQP5,** BCL6, **C1QTNF4,** CYP11A1, **DAPK3,** GATA3, **GLP2R,** HSD11B1, IL24, IL1A, LOX, **LRRC8A, MTL5, MZB1, NHLH1,** NR3C1, POMC, PPP3R1, PTX3, **RELT, RHCG,** S100A9, **SEMA5B,** SERPINA1, SFTPA1, SFTPC, **SIX1,** TAC1, TNFRSF11B, TRAF3IP2, **TRIM45, UMOD,** VDR	9	16	Organ Morphology, Endocrine System Development and Function, Lipid Metabolism

Supplement Table S4

Table S2. Primer sequences for qPCR

gene	Accession No.	primer	Amplified size
Mgat1		F: accattctgccagtgtttcc<br>R: caatatgccccgaacatacc	150 bp
Mgat2		F: aggagtgtcctgggtgtgac<br>R: tgatatgcatctcgggtcaa	145 bp
Mgat3		F: tccgctacttgctcgaaaat<br>R: ggcttagccatcatctcagc	173 bp
Mgat4a		F: gggatcccaaggagagagtc<br>R: tccaggatcatccagtcctc	190 bp
St3gal1		F: gacagtccacaacgctctga<br>R: caacccaccaacctcttgtt	159 bp
St3gal4		F: tatttgagttggccctgtcc<br>R: aggggatgccacagtaacag	212 bp
St3gal6		F: attgcctggttgtggaagtc<br>R: gcctgcatgggctacatagt	192 bp
Galectin3		F: agcccaacgcaaacagtatc<br>R: tctttcttcccttccccagt	143 bp
Galectin5		F: gaaggctgaggaaccctacc<br>R: gggtgtgaattgggaatgtc	129 bp
Galectin8:		F: aagatgtcggcacatcatca<br>R: tcctaaaccctggcaaaatg	240 bp
GAPDH		F: gtggacctcatggcctacat<br>R: tgtgagggagatgctcagtg	148 bp

F and R represent forward and reverse, respectively

Supplement Figure S1

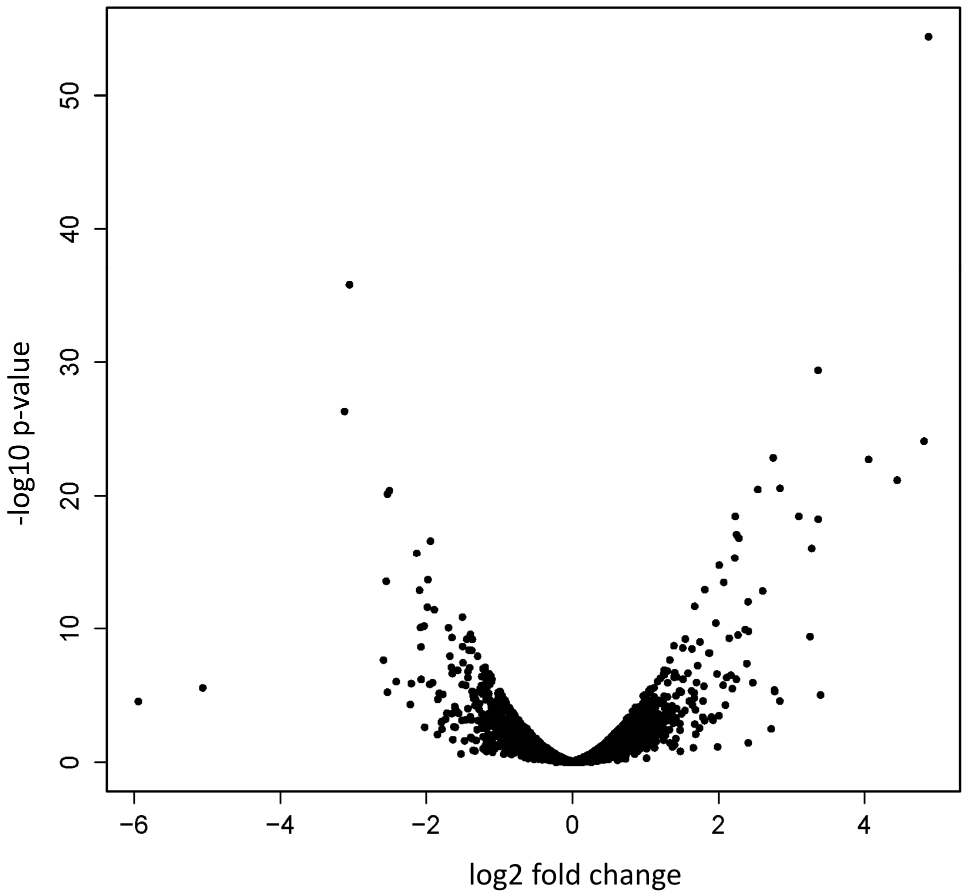

Supplement Figure S2

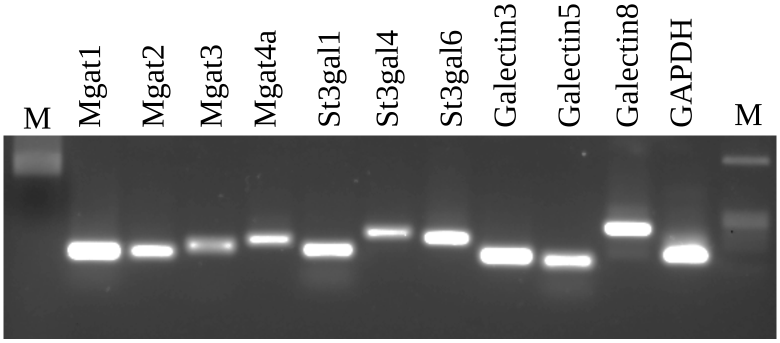

Supplement: Supplemental Table S1 — Summary of RNA-Seq coverage data. [file Table1.PDF]
